# Time-resolved force distribution analysis

**DOI:** 10.1186/2046-1682-6-5

**Published:** 2013-05-01

**Authors:** Bogdan I Costescu, Frauke Gräter

**Affiliations:** 1 Heidelberg Institute for Theoretical Studies, Schloss-Wolfsbrunnenweg 35, 69118 Heidelberg, Germany; 2 CAS-MPG Partner Institute and Key Laboratory for Computational Biology, Shanghai Institutes for Biological Sciences, Chinese Academy of Sciences, 320 Yueyang Road, Shanghai 200031, China

## Abstract

**Background:**

Biomolecules or other complex macromolecules undergo conformational transitions upon exposure to an external perturbation such as ligand binding or mechanical force. To follow fluctuations in pairwise forces between atoms or residues during such conformational changes as observed in Molecular Dynamics (MD) simulations, we developed Time-Resolved Force Distribution Analysis (TRFDA).

**Results:**

The implementation focuses on computational efficiency and low-memory usage and, along with the wide range of output options, makes possible time series analysis of pairwise forces variation in long MD simulations and for large molecular systems. It also provides an exact decomposition of pairwise forces resulting from 3- and 4-body potentials and a unified treatment of pairwise forces between atoms or residues. As a proof of concept, we present a stress analysis during unfolding of ubiquitin in a force-clamp MD simulation.

**Conclusions:**

TRFDA can be used, among others, in tracking signal propagation at atomic level, for characterizing dynamical intermolecular interactions (*e.g.* protein-ligand during flexible docking), in development of force fields and for following stress distribution during conformational changes.

## Background

Many biomolecular systems or other complex macromolecules can dynamically visit a broad range of conformational states. External perturbations such as a molecular interaction or a mechanical force can cause a molecule to dynamically transit between these conformational states. While the conformational space of biomolecules is typically analyzed by coordinate-based methods such as the detection of correlated motions, Force Distribution Analysis (FDA) has been recently developed as an alternative approach to analyze structure and structural transitions [1]. The advantages of analyzing internal forces instead of coordinates are two-fold. First, forces between atoms or residues represent internal coordinates, which consequently do not require any structural fitting. Secondly, forces are a more sensitive measure, as they are able to reveal low-amplitude yet functionally important motions such as those in a stiff protein core [2]. Among others, internal forces obtained from FDA proved able to explain the mechanical robustness of immunoglobulin domains [3] and to reveal the pre-stress in ubiquitin [4].

FDA has been applied so far to averaged dynamical data from Molecular Dynamics (MD) simulations. However, the dynamics of the force distribution within proteins or other macromolecules, *e.g.* in equilibrium, under a constant load, a load varying in time, or upon binding of another molecule, can only be characterized by following the changes of the internal forces in time, which cannot be easily achieved with the previous implementation of FDA. We here introduce a Time-Resolved Force Distribution Analysis (TRFDA) method, which adds a temporal component to FDA to enable the analysis of pairwise forces associated with conformational changes.

### Implementation

#### Overview

Time-Resolved Force Distribution Analysis (TRFDA) is based on the same concept of using pairwise forces as FDA, but focuses on their evolution during the MD simulation as well as on the analysis of large molecular systems. To achieve these goals, it stores in memory pairwise forces from only one integration time step at a time, such that the memory usage is independent from the length of the simulation. It also saves output data in new file formats, which are well suited for time series analysis. Similar to FDA, atomic pairwise forces are computed for all types of bonded interactions as well as from Coulomb and Lennard-Jones potentials; long-range electrostatic interactions computed on a grid (*e.g.* Particle Mesh Ewald [5,6]) cannot be decomposed in atomic pairwise forces. As in the previous FDA implementation, an MD simulation is first performed to obtain a trajectory, which is then used as the basis for a rerun using the TRFDA code. The core code is completely rewritten for GROMACS 4.5.3 [7], and VMD [8] plugins are provided for visualization. The implementation includes several notable improvements over FDA, which are discussed below.

#### Decomposition of forces from 3- and 4-body potentials

While two-body potentials such as bonds, Coulomb and Lennard-Jones potentials, can be directly used for the analysis of pairwise forces, many-body potentials which act on more than two atoms need to be decomposed into pairwise forces. TRFDA introduces a complete decomposition of the forces resulting from 3- and 4-body potentials (angle, dihedral angle, cross bond-bond, cross bond-angle), as described in the Additional file 1. The pairwise forces resulting from this way of force decomposition can have any direction, *i.e.* do not align with the vector connecting the two atoms involved. To accommodate this, all pairwise forces are stored and handled internally as vectors and are transformed into scalar values only when written to a file, if desired by the user.

The decomposition rectifies the shortcomings of the previous FDA code, which used approximations for computing pairwise forces resulting from 3- or 4-body potentials. For an angle formed by atoms *i*, *j* and *k* (*j* between *i* and *k*), it considered no pairwise forces to act on atom *j*, and assumed the pairwise force between atoms *i* and *k* to act along the distance vector between the two atoms. A similar decomposition has been used for a dihedral angle formed by four atoms. In contrast to these approximations, the decomposition used in TRFDA correctly reproduces the distribution of angle and dihedral forces in a molecule.

#### Internally computed pairwise forces between residues

Computing pairwise forces between residues allows a significant decrease in storage and computational cost for analysis, while providing a mapping of the interactions on the primary and possibly also secondary structure of a protein. The examination of pairwise forces between residues can also be used as a tool in the development of residue-level coarse grained models [9]. The pairwise force representing the interaction between residues *ri* and *rj* acts on the centers of mass of the 2 residues and is calculated as:

(1) $${\vec{F}}_{\text{ri},\text{rj}}=\underset{i\in \text{ri},j\in \text{rj}}{\sum }{\vec{F}}_{\text{ij}}$$

where *i* is an atom of residue *ri* and *j* is an atom of residue *rj*, with *ri* and *rj* being different. TRFDA computes internally pairwise forces between residues. As a consequence, TRFDA has relatively small memory requirements and completely avoids writing out pairwise forces between atoms, if only forces between residues are of interest. This is a significant improvement over the previous implementation of FDA, which involved storing in memory a large number of pairwise forces between atoms and saving them in a large file, which is subsequently read by a standalone tool.

A further advantage of TRFDA is the equal treatment of atoms and residues with respect to the output options. For example, the same vector to scalar transformations can be applied to both pairwise forces between atoms and pairwise forces between residues, punctual stress (see below) can be calculated both per atom and per residue, and the output file formats are the same for data referring to atoms or to residues.

#### Summed versus detailed pairwise forces

In TRFDA, the force decomposition described above allows several pairwise forces to be computed for the same atom pair. For example, two atoms forming a bond might also form angles and dihedral angles with neighboring atoms, resulting in multiple forces between them from different potentials. For two atoms, it is most often interesting to calculate the total interaction between them, independent of the underlying energy potentials. Only few applications, like force field development, might require a differentiation based on the potentials, with an associated increase in memory usage and output file size. TRFDA accommodates both scenarios: in the first case, it computes a vector sum from all pairwise forces between the two atoms, while in the second case it computes separate vector sums for pairwise forces resulting from different types of potentials.

In contrast, due to the approximations used for 3- and 4-body interactions in the previous FDA version, the bonded pairwise force between two atoms results from exactly one type of interaction.

#### Choice of output

TRFDA comes with a wide range of output options. Although internally forces are calculated and stored only in vector form, TRFDA can output pairwise forces as vector or scalar values, and several derived quantities based on them.

A scalar pairwise force is computed as the magnitude of the vector pairwise force, same as in FDA, or as the magnitude of the pairwise force projected onto the distance vector between the two atoms, same as in [4]. In either case, the scalar value carries a sign indicating whether it is a repulsive (plus) or attractive (minus) force. If the angle between the vector pairwise force and the distance vector between the atoms is in the range (- *Π*/2, *Π*/2), the pairwise force is considered attractive. If the angle between the vector pairwise force and the distance vector between the atoms is in the range (*Π*/2, 3 *Π*/2), the pairwise force is considered repulsive. If the vector pairwise force is perpendicular to the distance vector, the pairwise force can be considered neither attractive nor repulsive and it is set to zero.

From the absolute values of the scalar pairwise forces acting on an atom, TRFDA can compute the sum and average as well as select the minimum or maximum. The sum of the absolute values of scalar pairwise forces acting on an atom *i*:

(2) $$S_{i}=\underset{j}{\sum }|F_{\text{ji}}|$$

measures the stress acting on that atom. It is reminiscent of the calculation of stress from continuum models used in mechanical engineering. We denote the stress defined in Eq. 2 as *punctual stress*, emphasizing the action of the force on a dimensionless point instead of an area, which is here ill-defined. As a sum of forces, the punctual stress is expressed in units of force. Such a deviation from the typical definition of stress is also found for the atomic virial stress [10], expressed in units of energy, and stems from the difficulty of defining geometrical properties (area for punctual stress or volume for virial stress) at atomic level. For systems in equilibrium, the sum of scalar pairwise forces from Eq. 2 can converge to non-zero averages over time; in contrast, the sum of vector pairwise forces acting on a single atom *i*, typically computed in MD simulations, averages to zero in the same conditions. As opposed to the analysis of pairwise forces, which gives a very detailed view of their distribution in or between molecules, the punctual stress expresses in a simple way where pairwise forces accumulate and allows the detection of atomic-level “hot-spots”.

For both pairwise forces and per atom quantities, the output consists of simple to parse text files. Their format allows an unlimited number of atoms, pairwise forces and simulation time steps, and is documented in the manual, which accompanies the code. We chose text as opposed to binary file formats to allow for loading the data in various analysis tools at the expense of the size of the files.

TRFDA can also write out scalar pairwise forces in the same format as FDA, preserving compatibility with the FDA R library [1]; however the results should be interpreted with care, as the 3- and 4-body interactions are expressed differently.

#### Internal organization

TRFDA defines two groups of atoms between which pairwise forces are computed. A pairwise force is only computed when the two atoms belong to different groups, while pairwise forces between atoms of the same group are not computed. This allows, for example, interactions between a protein and a ligand to be computed efficiently. If the interactions inside the protein and ligand are also of interest, the two groups should comprise the same atoms, namely those of both protein and ligand. This way of selection avoids the calculation of possibly unnecessary pairwise forces within parts of the molecules, in contrast to the previous implementation of FDA [1].

For internal storage of pairwise forces, FDA uses a square matrix-like structure for which the memory usage depends on the atom numbering in the molecular system. TRFDA replaces this structure with lists for a much more efficient memory usage, opening the possibility of calculating pairwise forces for significantly larger molecular systems. Using lists also allows storing, for the same two atoms, of separate pairwise forces from the different potentials, *i.e.* the detailed pairwise forces described above. Searching in lists is not as fast as the direct access in the matrix-like structure, but this is partly offset by the better locality of data access.

## Results and discussion

Edwards *et al.*[4] demonstrated the existence of pre-stress in protein structures. They identified pairs of residues which interact strongly in equilibrium MD simulations and suggested that these interactions form a network which stabilizes the molecular structure. Multiply connected nodes, *i.e.* residues with several pairwise interactions under pre-stress, act like hubs in this network, strengthening it.

**Figure 1:**
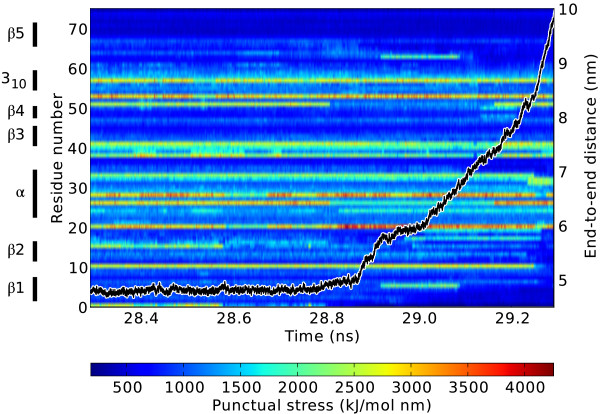
**Punctual stress during unfolding in a force-clamp MD simulation of ubiquitin.** The end-to-end distance (overlay black line) remains relatively constant until around 28.8 ns after which it increases abruptly, indicating the start of the unfolding process. Stress values are given at 1 ps time intervals, and have not been smoothed or averaged.

As a proof of concept, we here used TRFDA to follow the dynamics of the internal forces in ubiquitin during mechanical unfolding, as previously probed in single molecule force-clamp experiments [11]. Figure 1 shows the variation in time of the per residue punctual stress during force-clamp MD simulation of ubiquitin in water. Stress calculations were performed only on electrostatic forces, as those have been previously identified as the strongest links in the force network of ubiquitin [4]. A constant pulling force of 400 kJ/mol nm acts on the two termini and leads to protein unfolding after approximately 29 ns. Only the last 1 ns of the simulation is shown, during which ubiquitin unfolds, as reflected by the abrupt increase in the distance between the N- and C-terminal residues. The MD simulation was stopped upon reaching a 10 nm distance between the termini. The per residue punctual stress shows a very distinct pattern, with a few out of the 76 residues, primarily those involved in salt bridges and including those (Lys27, Asp52) identified in [4], featuring much higher stresses than most other residues.

Overall, the punctual stress fluctuates until around 28.6 ns suggesting that the network of pairwise forces is able to dynamically balance its load to resist the pulling force. We note that fluctuations in individual pairwise forces are large, but their time averages have been previously shown to converge on the nanosecond time scale of typical MD simulations (Figure 5 of [12]); the sum of their magnitudes, *S*_*i*_ in Eq. 2, varies much less in comparison. At a later time, significant variations in stress for many of the residues correlate with the stretching and unfolding of the structure. The stress gradually disappears from the *β*1 (Met1 - Thr7) strand and later the *β*2 strand, as they sequentially detach from the core of the protein during the unfolding process. The observed correlation in stress between the *β*1 and *β*5 strands reflects the concurrent rupture of these two strands from the protein core. The stress on several residues (Asp21, Lys29, Arg54, Asp58) remains high throughout the unfolding process shown in Figure 1, suggesting that the interactions in which these residues are involved survive until late in the unfolding process.

## Conclusion

TRFDA opens the possibility of following the evolution of pairwise forces during the course of an MD simulation, bringing insights into dynamic processes like transitions between stable states or signal transmission. It contains a force decomposition scheme for 3- and 4-body potentials, and can accurately represent the distribution of a pulling force in the molecular structure. Its output ranges from very detailed atomic pairwise forces, separated by the interaction potential and useful in force field development, to summed pairwise forces between residues used to map interactions to the protein structure. TRFDA can calculate a per atom or per residue punctual stress, which highlights the points where forces accumulate in a molecular structure, thereby pinpointing important elements which contribute to the mechanical resistance of the structure. Even though pairwise forces or stresses within a molecular structure can currently not be assessed by experimental means, we believe these observables to prove useful in analyzing and understanding the mechanical response of a complex molecule like a protein, just as stress calculations in structural mechanics analyses of macroscopic objects proved useful in assisting the design process. While atomic-force microscopy experiments and MD simulations of protein unfolding allowed unprecedented insight into the mechanical stability and rupture mechanisms of proteins, TRFDA enables revealing the underlying molecular basis of the mechanical response observed. The implementation focuses on computational efficiency and low memory usage, making it suitable for the analysis of large molecular systems and long MD simulations.

## Methods

The data used for the punctual stress analysis was ob- tained as follows:

All calculations were run with GROMACS 4.5.3 [7] using the OPLS-AA [13] force field. The ubiquitin structure 1UBQ [14] was solvated in a box of SPCE [15] water of 15x5x5 nm, and Na ^+^ and Cl ^−^ ions were added to obtain a 0.1 M salt concentration. A steepest descent minimization was carried out until the maximum atomic force was below 1000 kJ/mol nm. The molecular system was then equilibrated with a 0.1 ns NVT simulation followed by a 1 ns NpT simulation; during both these simulations, the heavy atoms of the protein were subjected to position restraints of 1000 kJ/mol nm. The short-range neighbor list, electrostatic and van der Waals cutoffs were set to 1 nm; long range electrostatic interactions were computed with the Particle Mesh Ewald method [5,6] using a FFT grid spacing of 0.16 nm. To allow an integration time step of 2 fs, all bonds were constrained using the LINCS algorithm [16,17]. At the beginning of the NVT simulation, random velocities were generated based on a Boltzmann distribution corresponding to a temperature of 300 K; the temperature was then maintained at 300 K by separate coupling of protein and water plus ions to a velocity rescaling thermostat [18] with a time constant of 0.1 ps. During the NpT simulation, the molecular system was coupled to an isotropic Parrinello-Rahman barostat [19,20] with a time constant of 2 ps and a reference pressure of 1 bar.

To unfold the protein, a force-clamp MD simulation was then performed in the same conditions as above, except that only the bonds between heavy atoms and hydrogen atoms were constrained to their equilibrium values. A constant force of 400 kJ/mol nm was applied to the C _*α*_ atoms of the C- and N-terminal residues in the direction of the largest dimension of the water box; the simulation was stopped when the distance between the C _*α*_ atoms exceeded 10 nm.

TRFDA was carried out on the trajectory obtained from the force-clamp MD simulation. Residue stresses were obtained according to Eq. 2, with only Coulomb interactions taken into account.

## Availability and requirements

*Project name:* TRFDA GROMACS *Project home page:*http://code.google.com/p/force-distribution-analysis/*Operating system(s):* Linux / Unix *Programming language:* C, additional VMD plugins in Tcl *License:* GNU GPL v2 *Any restrictions to use by non-academics:* none

## Competing interests

The authors declare that they have no competing interests.

## Authors’ contributions

BC developed and tested the software, analyzed the data, and wrote this manuscript. FG designed the experiment, and wrote this manuscript. Both authors read and approved the final manuscript.

## Supplementary Material

Additional file 1 **Multibody force decomposition.** Force decomposition for 3- and 4-body atomic interaction potentials. Click here for file
