# Supplementary material for: Time-resolved force distribution analysis
Source: BMC Biophys. 2013 May 1;6:5. doi: 10.1186/2046-1682-6-5 (PMC3669045; doi:10.1186/2046-1682-6-5)
Supplement: Additional file 1 — Multibody force decomposition. Force decomposition for 3- and 4-body atomic interaction potentials. [file 2046-1682-6-5-S1.pdf]

Time-resolved force distribution analysis
Additional file 1

Bogdan I. Costescu[1] and Frauke Gräter*[1,2]

[1]Heidelberg Institute for Theoretical Studies, Schloss-Wolfsbrunnenweg 35, 69118 Heidelberg, Germany
[2]CAS-MPG Partner Institute and Key Laboratory for Computational Biology, Shanghai Institutes for Biological Sciences, Chinese Academy of Sciences, 320 Yueyang Road, Shanghai 200031, China

Email: Frauke Gräter*- frauke.graeter@h-its.org;

*Corresponding author

Notation

$\vec{F_i}$ – force on atom i as vector

F_i – magnitude of $\vec{F_i}$

$\vec{U_i}$ – unit vector in the direction of $\vec{F_i}$

such that $\vec{F_i} = F_i \cdot \vec{U_i}$.

Forces decomposition for 3-body potentials

For an angle formed by atoms i, j, k (bonds i-j and j-k, see Figure 1), the sum of the atomic forces is zero:

$$\vec{F_i} + \vec{F_j} + \vec{F_k} = 0 \tag{1}$$

The atomic forces $\vec{F_i}$ and $\vec{F_k}$ can be decomposed into a component in the direction of $\vec{F_j}$ and a perpendicular component. The perpendicular components cancel each other out because of Equation 1. The components in the direction of $\vec{F_j}$ can be written as:

$$\vec{F_{ij}} = -\vec{F_{ji}} = F_i \cos(\vec{F_i}, -\vec{F_j}) \cdot \vec{U_j} \tag{2}$$

$$\vec{F_{kj}} = -\vec{F_{jk}} = F_k \cos(\vec{F_k}, -\vec{F_j}) \cdot \vec{U_j} \tag{3}$$

The pairwise force between atoms i and k can be obtained by a vector difference:

$$\vec{F_{ki}} = \vec{F_i} - \vec{F_{ji}} = -\vec{F_{ik}} = -(\vec{F_k} - \vec{F_{jk}}) \tag{4}$$

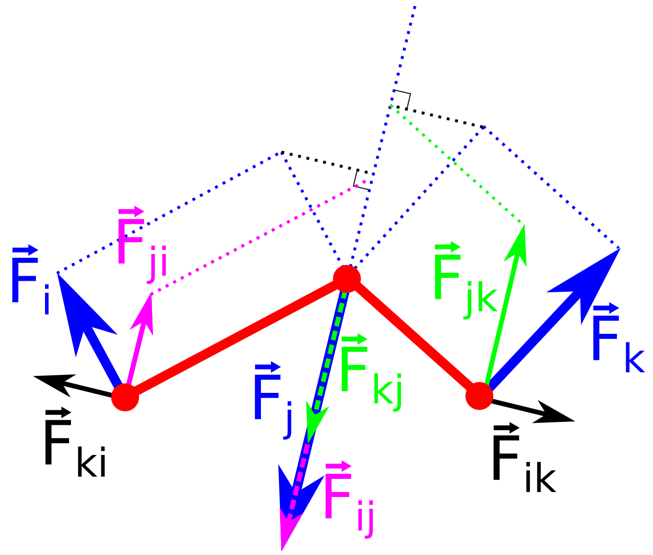

Figure 1: Force decomposition for an angle potential. $\vec{F_i}$ and $\vec{F_k}$ are decomposed into a component in the direction opposite to $\vec{F_j}$ and a component in the perpendicular direction.

Forces decomposition for 4-body potentials

For a dihedral angle formed by atoms i, j, k, l (bonds i-j, j-k and k-l, see Figure 2), the sum of the atomic forces is zero:

$$\vec{F_i} + \vec{F_j} + \vec{F_k} + \vec{F_l} = 0 \tag{5}$$

which can also be written as:

$$\vec{F_i} + \vec{F_l} = -(\vec{F_j} + \vec{F_k}) \tag{6}$$

Atoms j and k can be considered to form a single body on which a combined force:

$$\vec{F}_{j+k} = \vec{F_j} + \vec{F_k} \tag{7}$$

acts as shown in Figure 2a. Similar to the force decomposition for an angle, $\vec{F_i}$ and $\vec{F_l}$ can be decomposed in a component in the direction of $\vec{F}_{j+k}$ and a component in the perpendicular direction. The components in the perpendicular direction cancel each other out because of Equation 6. The components in the direction of $\vec{F}_{j+k}$ can be written as:

$$\vec{F}_{i(j+k)} = F_i \cos(-\vec{F_i}, \vec{F}_{j+k}) \cdot \vec{U}_{j+k} \tag{8}$$

$$\vec{F}_{l(j+k)} = F_l \cos(-\vec{F_l}, \vec{F}_{j+k}) \cdot \vec{U}_{j+k} \tag{9}$$

The pairwise forces acting on atoms j and k can be written in a generic way as:

$$\vec{F}_j = \vec{F}_{ij} + \vec{F}_{kj} + \vec{F}_{lj} \tag{10}$$

$$\vec{F}_k = \vec{F}_{ik} + \vec{F}_{jk} + \vec{F}_{lk} \tag{11}$$

and, because $j{+}k$ is considered a single body, j and k do not move with respect to each other, so the pairwise force between them is zero:

$$\vec{F}_{kj} = -\vec{F}_{jk} = 0 \tag{12}$$

such that:

$$\vec{F}_{j+k} = \vec{F}_{ij} + \vec{F}_{lj} + \vec{F}_{ik} + \vec{F}_{lk} \tag{13}$$

A decomposition of $\vec{F}_{i(j+k)}$ and $\vec{F}_{l(j+k)}$ can be made in the direction of $\vec{F}_j$ and $\vec{F}_k$, as shown in Figure 2b, such that:

$$\vec{F}_{i(j+k)} = \vec{F}_{ij} + \vec{F}_{ik} \tag{14}$$

$$\vec{F}_{l(j+k)} = \vec{F}_{lj} + \vec{F}_{lk} \tag{15}$$

Defining α as the angle between $\vec{F}_{j+k}$ and $\vec{F}_j$ and β as the angle between $\vec{F}_{j+k}$ and $\vec{F}_k$, these vectors can be written as:

$$\vec{F}_{ij} = -\vec{F}_{ji} = \frac{F_{i(j+k)} \sin\beta}{\sin\alpha\cos\beta + \sin\beta\cos\alpha} \cdot \vec{U}_j \tag{16}$$

$$\vec{F}_{lj} = -\vec{F}_{jl} = \frac{F_{l(j+k)} \sin\beta}{\sin\alpha\cos\beta + \sin\beta\cos\alpha} \cdot \vec{U}_j \tag{17}$$

$$\vec{F}_{ik} = -\vec{F}_{ki} = \frac{F_{i(j+k)} \sin\alpha}{\sin\alpha\cos\beta + \sin\beta\cos\alpha} \cdot \vec{U}_k \tag{18}$$

$$\vec{F}_{lk} = -\vec{F}_{kl} = \frac{F_{l(j+k)} \sin\alpha}{\sin\alpha\cos\beta + \sin\beta\cos\alpha} \cdot \vec{U}_k \tag{19}$$

Finally, the pairwise force between atoms i and l can be obtained by a vector difference:

$$\vec{F}_{li} = \vec{F}_i - \vec{F}_{ji} - \vec{F}_{ki} = -\vec{F}_{il} = -(\vec{F}_l - \vec{F}_{jl} - \vec{F}_{kl}) \tag{20}$$

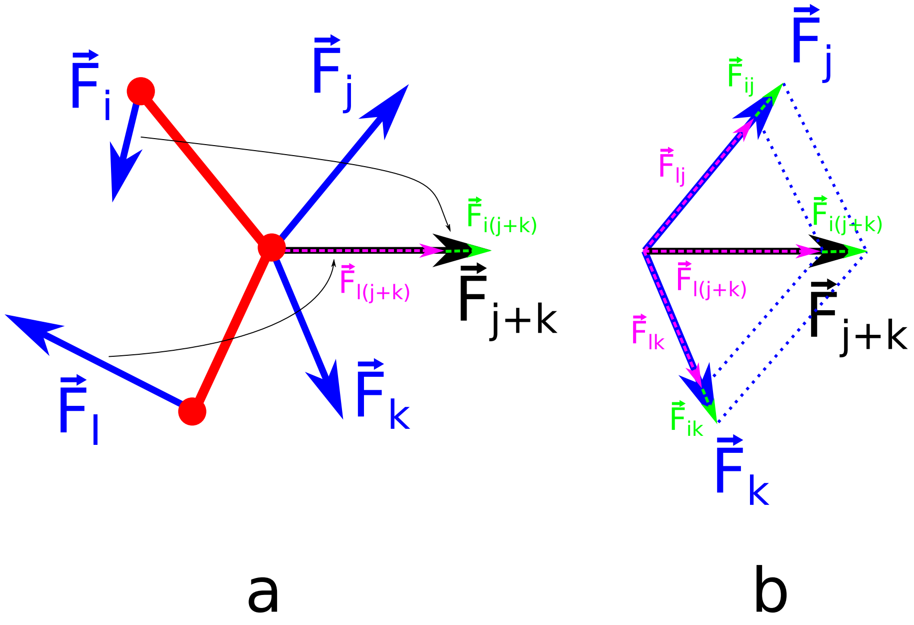

Figure 2: Force decomposition for a dihedral angle potential. a) Atoms j and k are considered a single body on which a combined $\vec{F}_{j+k}$ acts. Forces are then decomposed similar to Figure 1. b) The pairwise forces between the atoms i and l and the $j+k$ single body are decomposed into components in directions of $\vec{F}_j$ and $\vec{F}_k$.
